# Strategies for Controlling the Sporulation in *Fusarium* spp.

**DOI:** 10.3390/jof9010010

**Published:** 2022-12-21

**Authors:** Maria Ajmal, Adil Hussain, Asad Ali, Hongge Chen, Hui Lin

**Affiliations:** 1 College of Life Sciences, Henan Agricultural University, 95 Wenhua Road, Zhengzhou 450002, China; 2 Department of Entomology, Abdul Wali Khan University Mardan, Mardan 23200, Pakistan

**Keywords:** *Fusarium*, sporulation, anti-sporulation, spore inhibition

## Abstract

*Fusarium* species are the most destructive phytopathogenic and toxin-producing fungi, causing serious diseases in almost all economically important plants. Sporulation is an essential part of the life cycle of *Fusarium*. *Fusarium* most frequently produces three different types of asexual spores, i.e., macroconidia, chlamydospores, and microconidia. It also produces meiotic spores, but fewer than 20% of Fusaria have a known sexual cycle. Therefore, the asexual spores of the *Fusarium* species play an important role in their propagation and infection. This review places special emphasis on current developments in artificial anti-sporulation techniques as well as features of Fusarium’s asexual sporulation regulation, such as temperature, light, pH, host tissue, and nutrients. This description of sporulation regulation aspects and artificial anti-sporulation strategies will help to shed light on the ways to effectively control *Fusarium* diseases by inhibiting the production of spores, which eventually improves the production of food plants.

## 1. Introduction

*Fusarium* species are the most important phytopathogenic and toxic fungi distributed worldwide, and their spores act as infective propagules that initiate infection [1,2]. Fusaria are soil-born filamentous fungi, belonging to the class Ascomycetes and family Hypocreaceae. The genus *Fusarium*, which was characterized for the first time by Link in 1809, consists of hundreds of species, many of which are found in the soil, and some of them are associated with plants [3,4]. This fungus is found in tropical, subtropical, and also in temperate regions [5]. *Fusarium* produces white-, pink-, red-, purple-, salmon-, or grey-colored colonies with velvet to cottony surfaces. The capacity of this fungus to grow on a variety of substrates and its highly effective spore dispersal ability account for its extensive dissemination [6,7]. Therefore, understanding the strategies for the regulation and inhibition of the sporulation of *Fusarium* species is important for controlling their propagation and infection.

Some Fusaria are harmful to agricultural products, animals, and humans, because many of them are phytopathogenic and produce mycotoxins on plants that can adversely affect humans and animals if they enter the food chain [8,9,10]. *Fusarium* produces a variety of noxious secondary metabolites, such as fumonisins, zearalenone, and trichothecenes, that infect agricultural commodities and create a risk for human health and consumption [11]. The genome of *Fusarium verticillioides*, *Fusarium graminearum*, and *Fusarium oxysporum* f.sp. *lycopersici* contains about 46 secondary metabolite biosynthesis gene clusters that encode these mycotoxins [12]. There are more than 145 distinct *Fusarium* species, of which about one-seventh produce toxins [13]. As plant pathogens, *Fusarium* species result in significant economic damages and harvest losses [14].

*Fusarium* is one of the most economically destructive plant pathogens, causing major diseases in nearly all economically important plants and resulting in billion dollars of losses in the field of agriculture worldwide [15]. It is also capable of infecting crops in the moderate climate zones of the world. *Fusarium* produces mycotoxins such as *Trichothecenes* that can act as a source of infection in plant diseases [16,17,18,19,20,21]. As *Fusarium* is a soil-born plant pathogenic fungus, it can survive for a long time in soil by decomposing plant debris, the infected soil then moves from one place to another through animals or agriculture tools and can spread the pathogen to new areas [22]. Wilts, blights, rots, and cankers are diseases caused by *Fusarium* in many ornamentals, field crops, and forest trees [23]. The famous Panama disease of banana, also known as *Fusarium* wilt, was also caused by these fungi, which itself is one of the most destructive diseases of plants [5]. It is known from the literature that *Fusarium* diseases can survive in a variety of environmental conditions, but dry and warm weather is the most favorable condition for *Fusarium* wilt in chickpea [22,24].

Several cultural, physical, and chemical control strategies have been developed to control *Fusarium*. However, these strategies have little influence on *Fusarium* diseases, because Fusaria produce highly resistant chlamydospores, volatile inhibitors, and antibiotics [25]. Therefore, pathogenomics and biological control agents have gained great interest for the control of *Fusarium*. Various transgenic approaches have enabled the identification of genes, regulators, and transcription factors that are associated with virulence and pathogenicity. Fungal genes involved in pathogenicity may be employed as molecular tools for fungicide development or to develop transgenics [26,27].

## 2. Sporulation in *Fusarium*

Spores and fruiting bodies are the two most important morphological characters used by mycologists to categorize fungi into genera and to differentiate closely related species [28]. In fungi, asexual propagules are produced throughout the life cycle, typically requiring less investment for each propagule than for sexual spores, and dispersal is their sole function [29]. *Fusarium* species generate sexual spores and three different types of asexual spores. Less than 20% of *Fusarium* species, however, reproduce sexually, and not all *Fusarium* species produce all forms of spores [30]. As long as a food source is present, asexual spores are continuously produced in *Fusarium* and other fungi.

### 2.1. Sexual Spores

**Figure 1:**
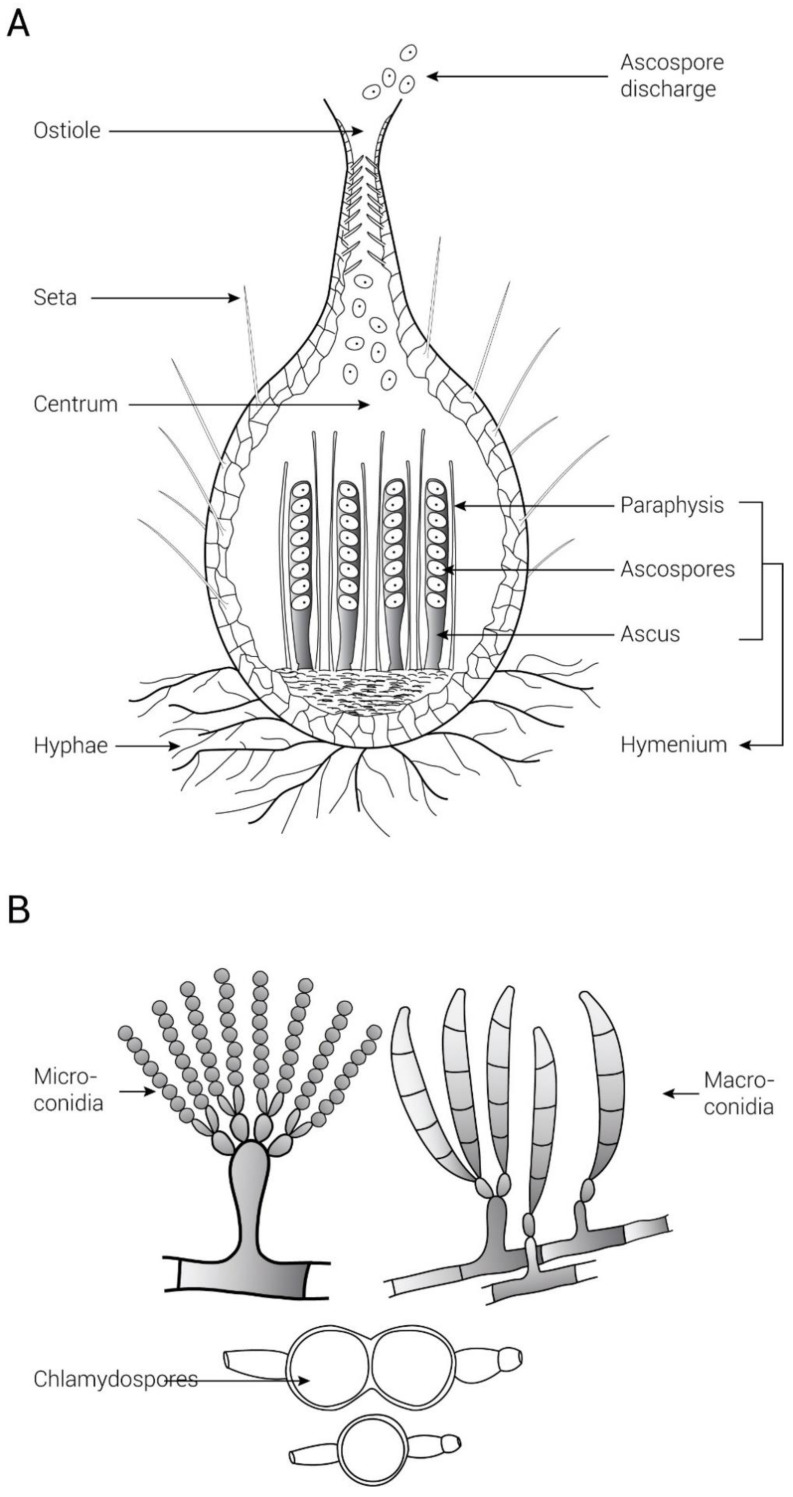
Sexual (**A**) and asexual (**B**) spores of *Fusarium*.

Some *Fusarium* species generate sexual spores (Figure 1). The role of sexual development in *Fusarium* spp. Is diverse. In *F. graminearum*, ascospore is an important primary inoculum that causes head blight disease of wheat and barley [31]. Furthermore, for disease production, sexual development was shown to be essential in *F. graminearum*, as it undergoes both sexual and asexual stages. In *Fusarim solani* f.sp. *pisi*, ascospores are not essential as propagules for dispersal [32]. As we know, the majority of *Fusarium* species produce fruiting structures in a culture (laboratory), but in the field, sexual development is rare. This is why out of the 12 species with *Gibberella teleomorphs*, fruiting is common only in *G. zeae*, whereas *G. fujikuroi* occasionally produce fruiting bodies in the field. The remaining species (*G. baccata*, *G. ircinate*, *G. coronicola*, *G. avenaceae*, *G. moniliformis*, *G. nygamai*, *G. pulicaris*, *G. intermedia*, *G. subglutinans*, and *G. thapsina*) have rarely or never produced fruiting structures in nature [33]. However, the majority of these species develop perithecia in the laboratory on natural or artificial substrates [3].

### 2.2. Asexual Spores

*Fusarium* species can produce three different forms of asexual spores (mitotic), including macroconidia, chlamydospores, and microconidia (Figure 1). These asexual spores are the most efficient means of reproduction and dispersal, and they also act as the main source of plant infection. These contagious propagules are crucial components of the disease cycle. They are also important for survival and protection in harsh environmental conditions [3,34,35]. Furthermore, chlamydospores play a vital role in the survival of *Fusarium* wilt diseases and thus cause more severe disease symptoms [36,37]. Chlamydospores are thick-walled cells that arise from mycelial hyphae, and conidia are produced in sporodochia, which are clusters of conidia-producing cells in a slimy mass [29]. Likewise, macroconidia are fusiform to sickle-shaped, multi-celled by transverse septa, with a foot-shaped basal cell. Microconidia can be globose, oval, and reni-form to fusiform, and they are often single-celled, though they can also be three- to five-celled. Only a few species generate microconidia in chains, but most do so in solitary or slimy heads [38]. Although *Fusarium* produces both sexual and asexual spores, asexual reproduction is more common. In this review, we focus on asexual reproduction.

### 2.3. Genetic Pathway Responsible for Spore Formation

With the advancement in molecular techniques, several genes in *Fusarium* that are involved in sporulation have been characterized. For example, in *F. graminearum*, several genes were identified and expressed that are reported to be involved in spore formation processes [39]. Similarly, in *Fusarium* and *Aspergillus*, mycotoxin production and sporulation are both regulated by G protein-signaling (RGS) pathways. Further, it was revealed that a number of genes were identified that are involved in the process of sporulation, altering several signal transduction pathway steps [40,41,42]. Furthermore, several regulators are also involved with asexual reproduction in *F. graminearum*. For instance, several genes required for conidiation are regulated by transcriptional factor *AbaA*, suggesting that *AbaA* is essential for asexual sporulation [43]. Meanwhile, in *F. graminearum*, *WetA* is required for conidiogenesis and maturation of the conidia [44]. 

Moreover, FgFlbA (RGS proteins) is required for conidiation in *Fusarium*, as it induces conidiation in *F. graminearum* [45]. For *F. graminearum* to produce asexual spores, a number of other proteins are also required. However, the appropriate expression of *HEX1*, which encodes the hexagonal peroxisome protein, is essential for controlling conidiogenesis [46]. Similarly, the autophagy-related lipase *Atg15* is also essential for morphogenesis and conidia formation [47]. In addition, *Mes1* (methyl salicylate esterase), a homologue of *MeSA*, is necessary for conidiogenesis in *F. graminearum* [48]. The deletion of velvet genes *veA* and *velB* showed increased conidial production [49,50,51]. Additional proteins involved in conidiation include *Mid1* (mating-induced death), *HDF1* (*histone deacetylase*), *CATs* (*carnitine acetyltransferases; CAT1 and CAT2*), *Acl* (*ATP citrate lyase*), and *Top1* (*topoisomerase I*) [52,53,54,55,56]. In a similar way, the actin binding protein and Fimbrin are also key factors in the conidiation process, as they increase the production of conidia in *F. graminearum* [57].

## 3. Growth Conditions and Environmental Factors Affecting Sporulation

Sporulation is mostly induced or stimulated by endogenous and environmental factors [40,58]. Environmental conditions that trigger sporulation include nutrient depletion, osmotic stress, oxidative stress, carbon and nitrogen status, calcium signaling, pH, aerial stimuli, desiccation, changes in CO_2_ partial pressure, secondary metabolites produced by competing organisms, and light. Similarly, endogenous factors such as conidiogenone, sporogen PF-1, and volatile organic compounds also stimulate conidiation [59,60,61]. However, various fungal species have diverse responses to these stimuli.

### 3.1. Temperature

**Figure 2:**
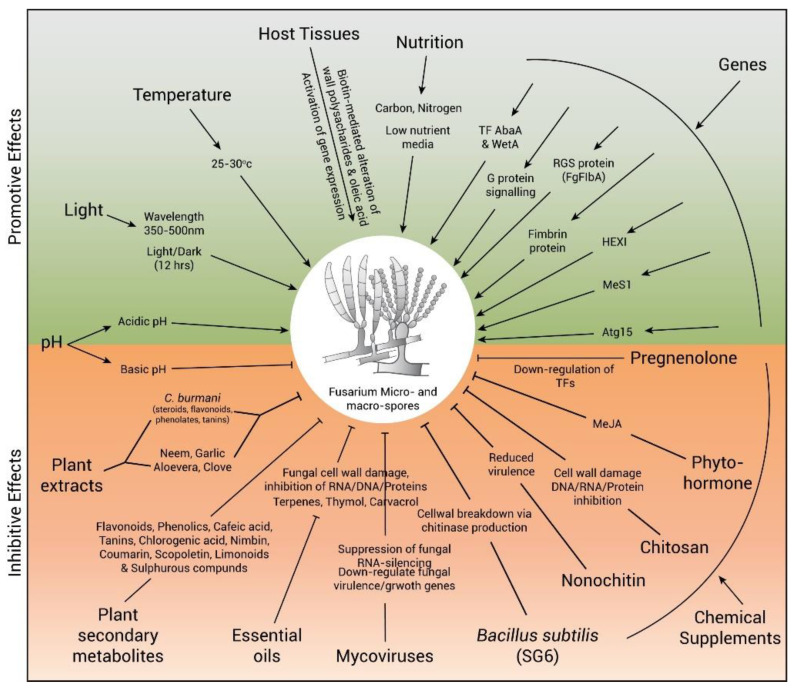
Factors affecting induction and inhibition of sporulation.

Temperature is an essential component that affects the growth and sporulation of *Fusarium* as well as the host’s susceptibility to diseases (Figure 2) [62,63,64]. Generally, *Fusarium* species can be grown in several temperature ranges [64,65,66,67]. However, the optimum temperature for growth and sporulation of *Fusarium* is 25–30 °C [68]. The optimal temperature for maximum growth and sporulation of *F. oxysporum* f.sp. *ricini* was shown to be 27 ± 2 °C on potato dextrose agar media [69].

### 3.2. Light

Light is considered to be one of the most important factors for spore formation (Figure 2). However, in some species of Basidiomycetes, Myxomycetes, Ascomycetes, and Zygomycetes, near-UV light irradiation successfully induced sporulation [70,71,72,73]. Excessive irradiation can inhibit sporulation. However, a wavelength ranging from 350–500 nm proved to be effective in enhancing sporulation [74,75,76]. For efficient sporulation, 12 h light and 12 h darkness is the best method available [77,78]. Under 12 h light and 12 h dark conditions, *F. solani* developed concentric sporulation ring patterns, but this pattern was lost when the fungus was exposed to continuous light or darkness [79]. Numerous other fungi, including *Fusarium*, were stimulated to sporulate by near-UV light, either on their own or in combination with cool white light [78,80]. Light also stimulated the production of metabolites in *Fusarium*, and in several other species, it also promoted the production of conidia and sexual fruiting bodies [81]. Several light-sensing proteins, such as White Collar-1 and the Vivid protein, and a few transcription factors, such as BLR-1 (blue-light regulator) and BLR-2, have been found to be involved in fungal sporulation [82,83,84,85,86,87,88,89]. Some *Fusarium* species also conidiate in the dark, rather than under continuous illumination, such as *Fusarium fujikuroi* [90].

### 3.3. PH

*Fusarium* mostly need various pH values for growth and sporulation (Figure 2) [91,92]. An acidic pH is most suitable for the growth and sporulation of *F. oxysporum* and *F. solani* [93]. *G. fujikuroi* and *F. oxysporum* were shown to grow and sporulate at 5–5.5 pH [94,95]. The best pH for the growth and sporulation of *F. oxysporum* was proved to be 5.5 to 7 [91,92,96]. 

### 3.4. Host Tissue

In pathogenic fungi, host tissues may also be used to stimulate sporulation (Figure 2). Banana petioles were shown to increase the sporulation of endophytic fungi isolated from wild banana (*Musa acuminata*) leaves [97]. The leaves of *Rhododendron pulchrum* cv. Ohmurasaki were autoclaved and used to enhance the sporulation of *Guignardia endophyllicola* [98]. Similarly, the leaves of *Dianthus caryophyllus* were also reported to be effective for conidiation in *Fusarium* and *Pestalotiopsis* species [99,100,101]. Though some plant tissues were also observed to be effective in inducing sporulation, such as in some *Botryosphaeriaceae* spp., pycnidia were stimulated using autoclaved pine needle [70]. Autoclaved corn hulls promoted macroconidia and mycelial growth of *F. graminearum*. Wheat bran and carnation leaves induced mycelial growth and macroconidia in *F. graminearum* and *F. proliferatum* [102].

Biotin also plays important role in the sporulation process. Due to its presence in plant tissue, it might change the formation of the cell walls and oleic acid, altering the expression of the genes related to sporulation [103,104,105,106,107]. Similarly, in mulberry leaves, biotin enhanced the sporulation of *Colletotrichum dematium* [108].

### 3.5. Nutrition

Some nutritional factors such as microelements, carbon, and nitrogen sources also influence sporulation (Figure 2) [109]. Therefore, several fungi need a particular amount of carbon and nitrogen for sporulation [110,111]. Moreover, sporulation is induced with reduced mycelial growth, and it is inhibited under factors that promote rapid mycelial growth [70]. Hence, food shortage or low nutrient media enhance sporulation [112,113]. Synthetic nutrient-poor agar medium, water agar media, and half- or ¼-strength potato dextrose agar (PDA) are some low-nutrient media that induce sporulation [114]. The polysaccharides starch and inulin were shown to induce sporulation in *F. oxysporum*.

For fungal isolation and culture, PDA is the most commonly used medium. Similarly, potato sucrose agar, Czapek yeast autolysate agar, yeast extract-phosphate medium, cornmeal agar, malt dextrose agar, V8 vegetable juice agar, potato carrot agar, and malt extract agar are also widely used mediums [115]. These media promoted the growth of many endophytic and pathogenic fungi, but they were not very effective in enhancing the sporulation of sterile isolates [116,117]. Furthermore PDA, MEA, and oatmeal agar were shown to be the best mediums for the induction of sporulation in *Fusarium* [93,118,119]. Similarly, MB and PDB media also promoted sporulation in *Fusarium* [93].

## 4. Artificial Control of Sporulation in *Fusarium*

*Fusarium* diseases are a major interruption to food production and are very difficult to control [120]. Farmers still use synthetic fungicides to control *Fusarium* disease. There are several other reasons to completely stop or minimize the use of synthetic chemicals, aside from their negative impact on the environment.

### 4.1. Biological Control Agents

Nowadays, botanical fungicides are used instead of synthetic fungicides for safety considerations. The botanical fungicides are developed from the extracts of higher plants, and these plant extracts contain antifungal and anti-microbial compounds that act as an anti-sporulation agent to control fungal diseases (Figure 2). In Indonesia, 37,000 plant species have been identified, but only 1% of them have been used as botanical fungicides [121]. Several tropical plant extracts possess antifungal activities that control plant pathogens [122,123,124,125]. Four species of plants, namely, *Eugenia aromatica*, *Piper bettle*, *Alpinia galanga*, and *Sphaeranthus indicus*, have been used as antifungal agents to control *F. oxysporum* f.sp. *vanilae* [126]. Similarly, the extracts of 14 tropical plants inhibited the growth of *F. oxysporum* f.sp. *capsici*, which causes *Fusarium* wilt in paprika [125]. Plant extracts of garlic, ginger, onion, neem, vinca, Indian pennywort, wild sage, marigold, and goat weed showed a complete inhibition of sporulation against *Fusarium moniliforme* [127]. Pea seed extract was used to inhibit the sporulation of *Fusarium oxysporum* f.sp. *pisi race2* [128]. Chinese gall was found to be effective in inhibiting the sporulation of *Fusarium graminearum*, and tillecur and white mustard seed flour were found to be best in inhibiting conidia in in vitro conditions [129]. Higher plants also produced secondary metabolites such as phenolic acid, cafeic acid, chlorogenic acid, and scopoletin, which are toxic to pathogens [130]. *Aloe vera* and clove plant extracts significantly inhibited the growth and spore formation of *F. oxysporum* f.sp. *lycopersici* [131]. Clove contains eugenol, and *Aloe vera* contains phenolic compounds as an antifungal agent [132,133].

#### 4.1.1. Leaf Extracts

The leaf extract of *Pometia pinnata* has been used to efficiently suppress potato late blight [134]. The leaf extract of *Cinnamomum burmanni* has been used to prevent the development of *Fusarium* wilt on tomato. It reduced the growth, biomass, and spore formation of *F. oxysporum* f.sp. *lycopersici*. The leaf extract of *C. burmanni* contains steroid, flavonoid, phenolate, and tannin, which are responsible for antifungal activity [135]. Similarly, leaf extracts of neem (*Azadirachta indica*) contain a highly toxic compound that showed a complete inhibition of sporulation and mycelial growth in *F. oxysporum* [136,137,138,139,140,141,142,143]. Neem contains antifungal compounds such as limonoids, protomeliacins, gedunin, azadirone, amino acids, vilasinin, salanin, nimbin, azadirachtin, coumarin., polysaccharides, sulphurous compounds, dihydrochalcone, glycosides, tannins, and flavonoids. These antifungal compounds are toxic and prevent the growth of pathogenic fungi [144,145,146,147,148,149]. The foliar spray of aqueous extract of neem showed antifungal activity against powdery mildew of balsam [150]. Neem was found to be best against tomato seedlings’ damping-off developed by *F. oxysporum* f.sp. *lycopersici* [151]. The leaf extract of *Parthenium hysterophorus* significantly suppressed the growth and spore formation of *F. oxysporum*, causing mung bean wilting [152].

#### 4.1.2. Essential Oils

Essential oil is also used as an antifungal agent against pathogenic fungi and is one of the most promising natural products for fungal inhibition. The main components of essential oil are carvacrol, thymol, and terpenes/terpenoids, which act as antifungal agents. The cell wall, cytoplasm, and mitochondria are the main targets for antifungal agents [153,154]. The antifungal agents can deactivate the fungus by disrupting the cell membrane and inhibiting the cell wall formation, the action of mitochondrial dehydrogenases, and efflux pumps. Because of their low molecular weight and high lipophilic nature, terpenes are capable of damaging the cell wall and cell membrane of fungi and also inhibiting its sporulation [154]. Similarly, the essential oil of *Litsea cubeba* contains citral, which acts as an antifungal agent against *F. moniliforme* and *F. solani*, affecting their cell wall and membrane, and it also inhibits DNA, RNA, and protein biosynthesis [155,156]. Garlic oil was shown to inhibit the mycelial growth and sporulation in *F. oxysporum*, which causes wilting in chili [137]. Other researchers have also used garlic against many diseases and reported that garlic contains a sulphur-containing antibiotic that is toxic to plant pathogens [157,158,159,160]. Garlic also contains allicin, which is the main antifungal compound [161,162]. Mint oil and clove oil reduced spore formation and the growth of *F. oxysporum* f.sp. *lycopersici* [131]. *Rosmarinus officinalis* essential oil reduced the sporulation of *F. verticillioides* [163].

#### 4.1.3. Mycovirus

Mycoviruses have also been used as natural enemies for the management of pathogenic fungi (Figure 2) [164,165,166,167]. These can trigger targets and in some cases suppress RNA silencing, which is the antiviral response of the fungus. Viruses defend themselves from the antiviral response of the fungus by suppressing RNA silencing. Mycoviruses regulate gene expression of the host fungus and also downregulate genes involved in virulence and growth. Wu et al. (2017) used the *Sclerotinia sclerotiorum 4* (SsMYR4) infection to downregulate the critical cellular activities and singling pathways of the host [168]. Moreover, the *F. graminearum* virus China 9 (FgV-ch9) and the *F. graminearum* viruses FgV1 and FgV2 induced hypovirulence in pathogenic fungi such as *F. graminearum* [169,170]. Thus, the relation of *F. graminearum* isolate china 9 with dsRNA mycovirus (Fgv-ch9) showed a significant reduction in conidiation [171]. In 2018, Lemus-Minor et al. used *F. oxysporum* f.sp. *dianthi* virus 1 (FodV1) to induce hypovirulence in *F. oxysporum*. This resulted in reduced mycelial growth, conidiation, and virulence on carnation plants, suggesting it functions as a biocontrol agent for *Fusarium* wilt of carnation [171].

#### 4.1.4. Rhizospheric Bacteria

Some rhizospheric bacterial species are employed as biological control agents, shielding plants from soil-borne diseases and promoting plant growth (Figure 2). *Streptomyces albospinus* CT205 and *Bacillus* sp. str. SV101 and SV104 have been used as biocontrol agents to inhibit *Fusarium* wilt [172,173]. In 2014, Zhao et al. used *Bacillus subtilis SG6*, which inhibits the growth and sporulation of *Fusarium graminearum*, to break down the cell wall of *F. graminearum* by producing chitinase [174]. *Paenibacillus polymyxa* NSY50 inhibited the growth of *F. oxysporum* in the rhizosphere of cucumber and thus protected the plant from pathogen invasion [175].

### 4.2. Chemical Supplements

Some chemical supplements are also used to control pathogenic fungi (Figure 2). Chitosan is known to inhibit spore formation and to act as antifungal agent. The fungal cell membrane is the primary target of chitosan [176]. The interaction of negatively charged phospholipid of the fungal cell membrane and positively charged chitosan increases membrane permeability that results in the leakage of cellular contents, which ultimately results in cell death [177]. They also function as chelating agents and bind to trace elements, hence rendering the vital nutrients inaccessible for the normal growth of fungi. Chitosan also punctures the fungal cell wall and binds to its DNA to inhibit the synthesis of mRNA [178,179]. Its inhibitory effect was proved with soil-borne phytopathogenic fungi, including *Fusarium* wilt pathogens [180,181,182]. It also inhibited the growth and sporulation of *F. solani* and *F. oxysporum* f.sp. *cubense* race 4 (FocR4) [183,184]. Nano chitin whisker also significantly inhibited the mycelial growth and conidiation of *Fusarium* species [185].

Potassium phosphonate inhibited the production of microconidia in *F. oxysporum* [186]. Similarly, pregnenolone inhibited sporulation in *Fusarium graminearum*. Pregnenolone might be targeted to the transcriptional factors required for sporulation [187]. Sulfamethoxazole and the indole alkaloid gramine are two natural compounds that decreased disease symptoms caused by *F. graminearum* in Arabidopsis and wheat [188].

Methyl jasmonate is a signaling molecule that modulates plant defense responses. It stimulates phenolic acids, flavonoids, and phytoalexins responsible for the plant’s defense against pathogens. [189,190,191,192]. Methyl jasmonate induced a defensin-like protein in *Pganax notoginsen* (*PnDEFL1*), which showed resistance to *F. solani* in transgenic tobacco [193]. Methyl jasmonate had an inhibitory effect on the sporulation and mycelial growth of *F. solani*. Radial growth and sporulation were significantly inhibited in *F. oxysporum* and *F. solani* by using different concentrations of salicylic acid [184,194]. Coumarin also inhibited the sporulation of *Fusarium oxysporum* f.sp. *niveum* by suppressing activities of pathogenesis-related enzymes [195].

### 4.3. Transgenic Approaches to Control of Sporulation in Fusarium

**Figure 3:**
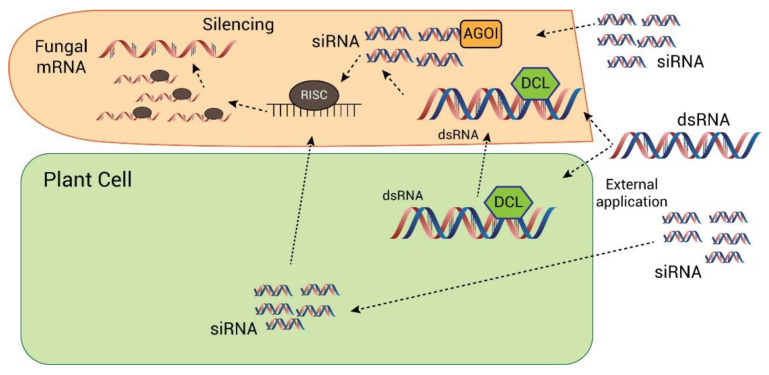
Long double-stranded RNAs (dsRNAs) are transferred to the plants. These are cut into small interfering RNAs (siRNAs) by fungal DCL proteins or plant Dicer-like proteins. The siRNA molecules bind to the complementary sequence of the target mRNA, resulting in its degradation.

For transgenic approaches to sporulation control in *Fusarium*, RNA interference (RNAi) is frequently used as a tool to regulate gene expression and provide protection against viruses and pathogens [196,197,198,199]. It was first reported in 1990 by Napoli and Jorgensen [200]. RNAi is activated in the presence of double-stranded RNA (dsRNA) in the host plant and degrades the double-stranded RNA molecule into single-stranded RNA molecules, hence causing silence or knockdown of the targeted gene of the pathogen. This artificial manipulation of gene silencing is used in both transgenic and non-transgenic plants and can be used to control *Fusarium* growth and sporulation by silencing the genes responsible for conidiation (Figure 3). Generally, there are two ways to perform RNA interference.

#### 4.3.1. Host-Delivered RNAi or Host-Induced Gene Silencing

Host-delivered RNAi (HD-RNAi) uses the host plant as a delivery system and silences the targeted gene of the pathogen [196,201]. In this approach, the siRNA or dsRNA is transformed to the host plant, thus targeting the gene of the pathogen. When this transgenic plant becomes infected, and the pathogen starts feeding from the host, the small interfering RNA (siRNA) and dsRNA molecules from the plant are transferred to the pathogen cells, hence activating an RNAi response in the pathogen and silencing the targeted gene of the pathogen [202]. This strategy was used on various *Fusarium* species. It was tested in tobacco against *F. verticillioides.* When the pathogen started feeding off transgenic tobacco plants, GUS-RNAi expressed and significantly silenced the *GUS* gene in the pathogen [203]. This technology was also used against *F. graminearum* in Arabidopsis and barley and significantly silenced three fungal cytochrome *P450 lanosterol C-14 α-demethylase* (*CYP51*) genes and also increased resistance against pathogens [204]. The silencing of the *Cmk1* gene (*Colletotrichum lagenarium* MAP kinase) in *C. lagenarium* showed a reduction in conidiation [205]. Similarly, *Fmk1*, *Hog1*, and *Pbs2* are mitogen-activated protein kinase genes responsible for fungal growth, development, sporulation, and virulence, and so the silencing of these genes in *F. oxysporum* showed reduced growth, sporulation, and pathogenicity [206]. However, silencing of the *Hog1* gene in *F. graminearum* showed significantly reduced conidiation [207]. Furthermore, the silencing of *FOW2* and *chsV* (class V chitin synthase) in *F. oxysporum* and *F. solani* showed reduced mycelial growth and sporulation, which confirmed their involvement in pathogenicity [208]. In *F. oxysporum* f.sp *cubense*, the *SGE1* gene (*Six gene expression 1*) is involved in pathogenicity and virulence. Therefore, silencing of this gene showed reduced sporulation and pathogenicity [209]. Moreover, the *ODC* gene (*ornithine decarboxylase*) in *F. oxysporum* is important for fungal growth and causes *Fusarium* wilt in tomato. Hence, the silencing of this gene showed resistance to *Fusarium* wilt in tomato [210].

#### 4.3.2. Spray-Induced Gene Silencing

The exogenous application of dsRNA and siRNA is another very promising approach to gene silencing [211,212]. The siRNA and dsRNA target the essential pathogen gene on the plant surface. They can also be sprayed on a wounded surface of the plant, and then this siRNA or dsRNA is taken up by the plants and transferred through the vascular system of fungi. This is an environmentally friendly strategy and is easily accepted by the public and biosafety authorities, and it is optimized faster than HIGS [213]. Koch et al. used this method on *Fusarium* and sprayed barley leaves with CYP3-dsRNA to check the growth of *F. graminearum*, and they found that the growth and conidiation of *F. graminearum* was inhibited by CYP3-dsRNA [214]. Myo5 dsRNA was sprayed on a wounded surface of the plant and silenced the Myo5 gene in the fungus. Myo5 has five segments, Myo5-3, Myo5-4, Myo5-5, Myo5-7, and Myo5-8, and all of these were significantly silenced by dsRNAs. As a result, both the sexual and asexual reproduction of *F. asiaticum* were significantly reduced. Meanwhile, Myo5-8 significantly reduced the growth of *F. asiaticum*, *F. tricinctum*, *F. graminearum*, and *F. oxysporum* f.sp. *lycopersici* [215].

## 5. Future Perspectives in Sporulation Control in *Fusarium*

*Fusarium* is one of the most harmful plant pathogens that causes wilt diseases of crops. *Fusarium* spores are easily spread in the field, causing invasive and disseminated infections. *Fusarium* sporulation is mostly induced or stimulated by endogenous and environmental factors. Several strategies have been developed to control the production of spores. In particular, various biocontrol agents and chemicals were used to control *Fusarium* sporulation, but most of these experiments were performed under in vitro conditions, so they should be validated under field conditions. We expect that more efficient biocontrol agents and chemicals will be identified from further field experiments. The management of *Fusarium* diseases by gene silencing was also considered to be a powerful method to control the sporulation of *Fusarium*, and more studies should be carried out in the future to characterize and identify the genes that are involved in sporulation. Currently, two genes in the ergosterol synthetic pathway that are relevant to the sporulation of *Fusarium* were identified by our team (unpublished). The control of the sporulation of *Fusarium* and then the control of the spread of wilt diseases will eventually become a new approach to increase crop yield and quality.

## Data Availability

The data presented in this study are available on request from the corresponding author.
